# Calibration‐Free GRAPE pTx Pulses for Homogeneous Spatial‐Selective Excitation at 7T

**DOI:** 10.1002/mrm.70266

**Published:** 2026-01-26

**Authors:** Daniel Löwen, Eberhard D. Pracht, Marten Veldmann, Vincent Gras, Franck Mauconduit, Nicolas Boulant, Tony Stöcker

**Affiliations:** ^1^ MR Physics German Center for Neurodegenerative Diseases (DZNE) Bonn Germany; ^2^ Université Paris‐Saclay Commissariat à l'Energie Atomique, CNRS, NeuroSpin, BAOBAB Gif sur Yvette France; ^3^ Department of Physics & Astronomy University of Bonn Bonn Germany

**Keywords:** GRAPE, parallel transmission, ultra‐high field, universal pulses

## Abstract

**Purpose:**

Extend the universal pulse GRAPE formalism to pulses with a defined spectral response, and apply the concept to spatial selection.

**Methods:**

We added Bloch simulations at several frequencies for each voxel to the pulse calculation to create universal spectrally‐selective GRAPE pulses. With a superimposed constant gradient field spatial selection was achieved. The method was tested in slice‐ and slab‐selective imaging experiments.

**Results:**

Universal spatially‐selective GRAPE pulses increased FA homogeneity and SNR. In 2D gradient echoes, the SNR could be increased by approximately 6% compared to CP pulses, and in a slab‐selective TSE sequence, the SNR increased by 29% against k
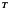
‐spokes pulses. Additionally, the slab‐selective GRAPE pulse proved to be more robust against $B_{0}$ deviations and is significantly shorter in comparison to k
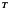
‐spokes pulses while maintaining a similar FA homogeneity.

**Conclusion:**

Spatially‐selective universal GRAPE pulses exhibit superior performance compared to k
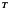
‐spokes pulses. These short and robust pTx pulses hold potential for enhancing a wide range of imaging applications, thereby advancing 7T MRI technology closer to clinical use.

## 1 Introduction

The popularity of ultra‐high field (UHF) MRI has increased significantly in recent years due to its inherently higher signal‐to‐noise ratio (SNR) compared to low and high field MRI [1, 2], enabling measurements in shorter acquisition times or with higher resolutions [3]. Despite remaining challenges there are already 7T scanners available for clinical use [4]. One of the main challenges is posed by increased radio frequency (RF) power deposition and RF transmit field ($B_{1}^{+}$) inhomogeneities [5, 6], which lead to signal loss or potentially contrast distortion. Both problems can be addressed by parallel transmission (pTx) coils, which allow multiple transmit channels to be driven independently [7, 8]. Extensive research has been conducted to develop coil technologies and pTx methods to improve the efficiency and precision of the excitation fields [9, 10].

The simplest approach to improve the transmit field homogeneity compared to commonly used circularly polarized (CP) pulses is $B_{1}^{+}$‐shimming [11]. Here, each transmit channel is assigned an optimized complex weighting factor while all channels share the same temporal RF waveform. This method allows quick subject‐specific $B_{1}^{+}$ adjustments within a few seconds and is integrated into most ultra‐high field MRI systems, but has limited capabilities in improving $B_{1}^{+}$ homogeneity.

Significantly improved homogeneity or dedicated excitation patterns can be achieved by providing channel‐specific temporal RF and magnetic field gradient waveforms [12]. A very effective and computationally efficient pTx method is k
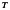
‐points pulses [13], which consist of a few square sub‐pulses. Each sub‐pulse assigns a different weighting factor to each transmit channel, and small gradient moments (“blips”) are applied between the sub‐pulses. With this approach, the RF waveform is sufficiently flexible to achieve homogenous excitation, but it is constrained to a fixed RF envelope for each sub‐pulse, such that computations can be completed in a reasonable time. The concept was first introduced for slice‐selective multi spokes pulses (“fast‐k
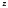
”) [14, 15], where each sub‐pulse has a sinc‐shaped envelop. Later, the concept was generalized for flexible slab positioning and orientation (“k
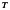
‐spokes”) [16, 17].

PTx solutions without any parameterization exploit the full potential of pTx but come with a large computational burden. Optimal control solutions were already proposed in 2004 using the gradient ascent pulse engineering (GRAPE) algorithm [18, 19] to design RF and gradient waveforms without any parametrization (“GRAPE pulses”). Yet, their usage is challenging for subject‐specific optimizations in a single scan session, since the complex computation may extend the exam duration to unacceptable lengths.

However, with the proposal of universal pulses (UP) [20], it was possible to use pTx pulses without any calibration scans and pulse optimizations during the actual imaging session. UP are designed using a database of previously acquired $\Delta B_{0}$ and $B_{1}^{+}$ maps from multiple subjects. It was shown that UPs are sufficiently robust against inter‐subject variability, such that homogeneous excitation can be achieved on new subjects not included in the database [21, 22]. When employing UPs, concerns about computational burden become less important, making GRAPE pulses more attractive [23].

To date, GRAPE pulses have been used mainly for 3D non‐selective excitation and refocusing [24] or for subject‐specific imaging at a predefined position [25]. The latter approach still requires two sessions because the pulse optimization time takes several hours. Therefore, we propose a method to design universal GRAPE pulses capable of slice‐ and slab‐selective excitations, while allowing flexible positioning of the field of view during the imaging session.

## 2 Methods

### 2.1 Spectral‐Selective GRAPE Pulses

Aside from $B_{0}$ offsets, constant magnetic field gradients establish a linear relationship between the spatial position and precession frequency [26]. An RF pulse with a sinc‐shaped envelope yields a rectangular spectral profile, which translates to a spatially‐selective rectangular excitation pattern. The location of the excited slice can subsequently be adjusted by altering the frequency of the RF pulse.

To extend this concept toward the design of spatial‐selective GRAPE pulses, it is necessary to ensure that the magnetization in all voxels exhibits the same spectral response. This requirement can be addressed during pulse optimization, where Bloch simulations are performed at each iteration for each voxel across a range of frequency offsets f with a frequency dependent target response. Frequency offsets can be randomly drawn from a Gaussian or uniform distribution, with each voxel assigned a unique set of frequency samples to prevent oscillations as described in [25].

However, evaluating Bloch simulations for every voxel across numerous frequency offsets at each iteration is computationally demanding. To reduce the computational effort, it is assumed that the $B_{0}$ and $B_{1}^{+}$ field maps vary smoothly across neighboring voxels. This spatial smoothness allows to keep the number of frequency evaluations per voxel small without significantly compromising accuracy. By exploiting this smooth variation, the computational load is substantially decreased while maintaining the fidelity of the optimization process.

### 2.2 Definition of the Cost Function

The target flip angle, denoted as ${\text{FA}}_{T}(f)$, is defined to be constant for all voxels within the pass‐band, that is, for frequency offsets satisfying $|f|\leq BW_{T}/2$, and zero for voxels outside:

(1) $${\text{FA}}_{T}(f)=\left\{\begin{array}{ll} {\text{FA}}_{T}\; & \text{if}\;\left|f\right|\leq BW_{T}/2\\ 0\; & \text{else} \end{array}\right.$$

A magnitude‐least‐squares (MLS) optimization would lead to arbitrary phase profiles φ(f) across frequency offsets, which, in the presence of a gradient field, translates directly into spatial phase variations along the slice‐selection direction. As a result, the final magnetization is not rephased uniformly, rendering such solutions possibly unusable for many imaging applications. It is therefore necessary to account for the phase of the magnetization in the optimization process. While a complex‐least‐squares (CLS) optimization enables explicit control over both magnitude and phase, it requires to specify a certain spatial phase profile. This imposes unnecessary constraints on the optimization, potentially leading to suboptimal results.

To enforce a linear phase across the excitation spectrum for each voxel without prescribing a specific spatial phase profile, a phase consistency term was added to the MLS cost function. For this purpose, the phase of the magnetization is computed at a given frequency offset f and at a slightly shifted offset f+Δf. The difference between these two phases, φ(f)−φ(f+Δf) is then constrained to match the phase evolution expected from free precession over half the duration of the RF pulse, that is, $\varphi (f)-\varphi (f+\Delta f)=\pi \Delta fT=\Delta \varphi_{T}$, where T denotes the total pulse duration.

The optimization process is initialized using a CP, sinc‐shaped RF pulse as the starting point. Subsequently, the pulse is optimized by minimizing the following cost function:

(2) $$c=\sqrt{\begin{array}{l} \sum_{f,\text{voxel}}w(\Delta B_{0},f){\left(\frac{\text{FA}(f)-{\text{FA}}_{\text{T}}(f)}{{\text{FA}}_{\text{T}}(f=0)}\right)}^{2}+\lambda_{\text{ph}}\sum_{\text{passband}}w_{\text{ph}}(\text{FA}(f))w(\Delta B_{0},f){\left(\frac{\varphi (f)-\varphi (f+\Delta f)-\pi \Delta fT}{\pi \Delta fT}\right)}^{2} \end{array}}$$

**FIGURE 1:**
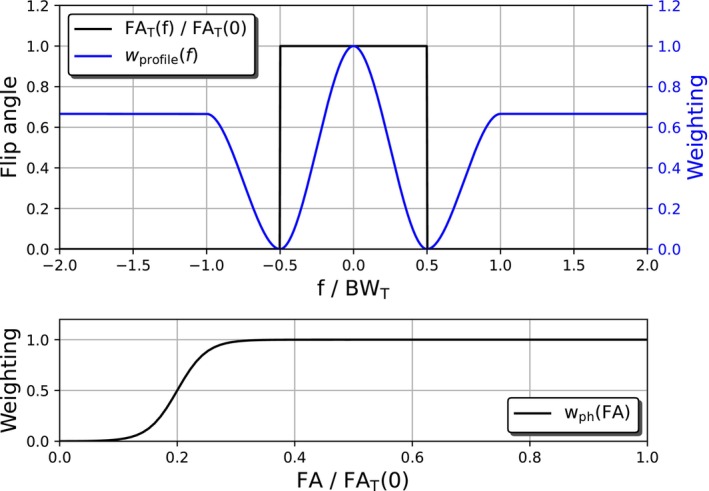
Profiles of the target flip angle ${\mathrm{FA}}_{T}(f)$ (top, black), the corresponding frequency‐dependent weighting term $w_{\text{profile}}(f)$ with respect to the frequency f (top, blue) and $w_{\mathrm{ph}}(\text{FA})$, which depends on the simulated flip angle (bottom).

The first term under the square root corresponds to an MLS objective, which enforces the target flip angle magnitude (as defined in [23]). The second term promotes a linear phase within the pass‐band. Both terms are weighted by a frequency‐ and voxel‐dependent weighting function defined as:

(3) $$w(\Delta B_{0},f)=w_{\text{profile}}(f)\cdot \left(1+\lambda_{B0}\left|\Delta B_{0}\right|\right)$$

where $\Delta B_{0}$ represents the static field deviation at a given voxel, $\lambda_{B0}$ is a constant scaling factor [23], and $w_{\text{profile}}(f)$ is a frequency‐dependent weighting term. The inclusion of $w_{\text{profile}}(f)$ is necessary because deviations from the target flip angle contribute quadratically to the cost function. In practice, the actual flip angle profile exhibits a smooth transition between the pass‐band and stop‐band, while the target is an ideal box function. Without appropriate weighting, discrepancies near the transition regions would disproportionately dominate the cost function. Therefore, $w_{\text{profile}}(f)$ is designed to decay to zero at the boundaries between pass‐band and stop‐band (cf. Figure 1 top), reducing the influence of these regions during optimization. Additionally, by adjusting $w_{\text{profile}}(f)$ the balance between stop‐band and pass‐band can be modified, such that side lobes are suppressed and the homogeneity in the pass‐band is as high as possible.

The second term, which addresses the phase behavior, is scaled by a factor $\lambda_{\text{ph}}$, allowing to balance the flip angle homogeneity against phase uniformity. Furthermore, another weighting function $w_{\text{ph}}(\text{FA}(f))$ is incorporated to reduce the influence of the phase term when the flip angle FA(f) approaches zero (cf. Figure 1 bottom). This prevents phase deviations in regions with small flip angles from impairing the optimization result. We chose the hyperbolic tangent, as a smooth and differentiable step function and shifted it by 20% of the target flip angle.

### 2.3 Pulse Design

All pulses were calculated using an in‐house built pulse optimization toolbox written in Python, which utilizes the sequential least squares programming (SLSQP) algorithm from Scipy [27] to minimize the cost function. The program's workflow matches that described in our previous work [28]. The optimization constraints were chosen according to the hardware limitations and SAR constraints, with a maximal gradient field strength of 70 mT/m, a gradient slew rate of 200 T/m/s, a peak voltage of 190 V, a total power of 8 W, and a power per transmit channel of 1 W. We found that pulses with symmetric RF and gradient waveforms performed similar to non‐symmetric pulses. Therefore, we decided to design symmetric pulses, which reduces the degrees of freedom in the optimization and speeds up the calculations.

Furthermore, the concept of UP [20] was applied to all pulses, allowing for calibration‐free application. Therefore, we used a database of 20 subjects, where $B_{0}$ maps were acquired with a 3D multiple gradient recalled echo (GRE) [29] and the complex sensitivity profiles of all eight channels of the head array coil were acquired by using the actual flip‐angle imaging sequence (AFI) [30].

#### 2.3.1 Slice‐Selection

To examine the potential of the new method for slice‐selective applications, we employed a 2D GRE sequence. The pulse properties were chosen to match the flip angle, duration, bandwidth, and energy of the vendor‐provided CP excitation pulse with a flip angle of 20°, duration of 3 ms and time bandwidth product of 10 (corresponding to a bandwidth of 3.3 kHz). Five frequency offsets were assigned to each voxel for the optimization and were randomly drawn from a Gaussian distribution with σ= 6.6 kHz, $\lambda_{B0}$ was set to 0.02 and $\lambda_{\text{ph}}$ to three. To speed up the optimization (resulting in a computation running for 5 days (120 h)), the pulses were optimized with an RF and gradient sampling time of 30μs and afterwards linearly interpolated to 10μs.

Two different strategies were used to design the GRAPE slice‐selective pulse. In the first strategy, a single pulse was optimized for the whole brain. This provides total flexibility with respect to the slice position during the imaging session (slice shifts and angulation). In the second strategy, individual pulses were optimized for each slice position covering the whole brain. For this purpose, the contribution of each voxel in the cost function was multiplied by a weighting factor $w_{\text{ROI}}$ that exponentially decreased with the distance d from the respective slice center ($w_{\text{slice}}=\exp(-d/10\;\text{mm})$) [31]. The slice orientation was chosen to be sagittal [32] and the position was determined with reference to the isocenter of the scanner, which was almost identical to the mid of the brain for each subject in the optimization database. With this approach, the pulses are limited to a fixed FOV position and orientation, but should provide enough flexibility due to the exponentially decreasing weighting function and the variance of head positions and tilts in the database. Since these pulses were optimized for a specific region of interest (ROI), they are denoted as “GRAPE slice‐selective ROI”. To reduce the computational burden, the optimizations were initialized with the result from Strategy 1 and pulses were only optimized for every second slice and the resulting pulses were also used for the respective adjacent slice.

#### 2.3.2 Slab‐Selection

To demonstrate slab‐selection, GRAPE pulses were designed for a single‐slab T
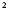
‐weighted turbo spin echo (TSE) sequence with a variable flip angle train (vFA) [33, 34]. The GRAPE pulse was compared to universal k
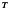
‐spokes pulses designed for this sequence in a previous work [28]. The sequence applies a slab‐selective excitation pulse, followed by a non‐selective 180° refocusing pulse and a subsequent non‐selective vFA refocusing pulse train [35, 36, 37]. Due to the relatively long slab‐selective k
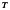
‐spokes excitation pulse (9.5 ms including rephasing gradient), it is necessary to use a non‐selective 180° refocusing pulse after the excitation, which allows the reduction of the echo spacing in the subsequent echo train [38].

In this work, we replaced the k
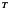
‐spokes pulse with a GRAPE slab‐selective excitation pulse. The pulse design properties were: ${\text{FA}}_{T}=90$, duration = 3 ms, sampling time = 20μ s (afterwards interpolated to 10μ s), $BW_{T}=2.5$ kHz, $\lambda_{B0}=0.01$, $\lambda_{\text{ph}}=3$ and three frequency offsets were assigned to each voxel drawn from a uniform random distribution inside the interval [−6.25, 6.25 kHz]. A uniform random distribution was chosen in this case, as signal at large frequency offsets would appear far outside the FOV and therefore can be neglected in the optimization. The optimization was running for seven days (168 h).

The refocusing pulses were designed exactly in the same manner for k
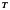
‐spokes and GRAPE excitation with the respective target phase to fulfill the Carr–Purcell–Meiboom–Gill (CPMG) condition [39, 40].

### 2.4 Experiments

Experiments were conducted on a MAGNETOM 7T Plus scanner (Siemens Healthineers, Erlangen, Germany) using a head array coil with 32 receive and eight transmit channels (Nova Medical, Wilmington, USA). In vivo experiments were performed in accordance with guidelines set by the institutional review board.

#### 2.4.1 Slice‐Selection

The computed slice‐selective GRAPE pulses were integrated in a 2D GRE sequence with a center‐out spiral readout and 1.5 mm slice thickness. The spiral readout was segmented into 33 spiral interleaves with an in‐plane acceleration factor of two. Other parameters were: 1.5 mm isotropic resolution, 102 slices in sagittal orientation, 210 x 210 mm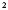 in‐plane FOV, TE = 5.22 ms, TR = 5.1 s, readout duration = 1.41 ms. The total acquisition time (TA) was 3:27 min with a 23 s Cartesian reference scan for parallel imaging in the beginning. Sensitivity maps were calculated with ESPIRiT [41] and images were reconstructed with the “pics” command of the BART toolbox [42]. The GRAPE pulses were compared to CP and (subject‐specific) $B_{1}^{+}$‐shimmed sinc pulses (the adjustment volume covered the whole brain). CP and $B_{1}^{+}$‐shimmed pulses were manually rescaled (by adjusting the reference voltage) to achieve the nominal flip angle. SNR maps were calculated with the pseudo‐replica method [43] using 100 replicas.

For slice profile measurements, an additional phase‐encoding gradient was added along the slice direction. The FOV along the slice direction was 3.2 mm with a resolution of 0.2 mm (16 phase‐encoding steps). The TE was increased to 6.65 ms, the in‐plane FOV was 5 mm and four spiral interleaves with an in‐plane acceleration factor of three were used. The TA time was 3:24 min. All other parameters were kept unchanged.

Images and slice profiles were acquired from two healthy male subjects. Additionally, $B_{0}$ and $B_{1}^{+}$ maps were measured for the corresponding simulations. In this case, the 3DREAM sequence [44] was used for $B_{1}^{+}$ measurements (4 mm isotropic resolution and TA=0:36 min), to reduce the total scan time.

For each slice, the flip angle in the center of the slice and the integral over the slice were calculated in Bloch simulations. The slice integral was calculated over 3000 spins in each voxel equidistantly arranged along a 6 mm line in slice selection direction. $B_{0}$ and $B_{1}^{+}$ maps were linearly interpolated for each spin position. Both flip angles and phases were taken into account to estimate the signal strength. A large deviation between the central FA and the slice integral would indicate unintentional phase variations along the slice‐selection direction, requiring to adjust $\lambda_{ph}$. Simulations were performed on 10 subjects not included in the optimization database.

#### 2.4.2 Slab‐Selection

The slab‐selective vFA TSE sequence was set up with the following parameters: TR/TE (TE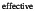) = 4000/379 (118) ms, Echo spacing = 6.65 ms, 1.4 averages, 56 slices per slab, 28.6% slice oversampling, $R_{PI}$=1x3z1 (CAIPIRINHA) [45], readout pixel bandwidth = 258 Hz/px, turbo factor = 107, voxel size = 0.45 x 0.45 x 1.0 mm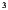, FOV = 224 x 182 x 56 mm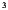, TA = 6:48 min. SNR maps were calculated with the pseudo‐replica method using 100 replicas.

GRAPE pulses were compared to k
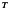
‐spokes pulses and $B_{1}^{+}$‐shimmed sinc pulses (the adjustment volume covered the selected FOV).

**FIGURE 2:**
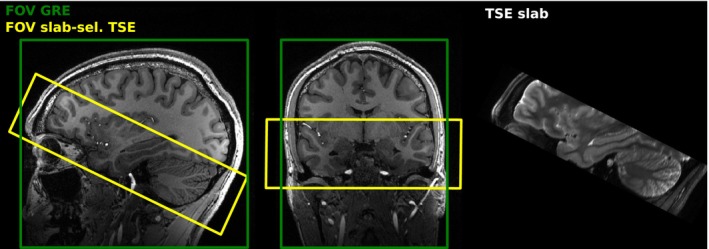
The field of view (FOV) for the GRE (green) and the TSE (yellow) acquisition are illustrated on top of a T
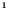
‐weighted image. The slab‐selective TSE was acquired to cover the frontal lobe, the temporal lobes and most of the cerebellum. On the right side, the acquired slab in sagittal orientation is illustrated.

The FOV was determined based on two primary considerations. First, the cerebellum was included as this region exhibits the lowest $B_{1}^{+}$ values when using conventional CP pulses. Second, the FOV covered the inferior portions of the frontal and temporal lobes, where adjacent air‐filled cavities (such as the frontal sinuses and external auditory canals) cause substantial $B_{0}$ inhomogeneities. Consequently, an oblique slab orientation was selected, as depicted in Figure 2.

T
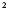
‐weighted images, $B_{0}$ and $B_{1}^{+}$ maps were acquired from two healthy male subjects. Additionally, simulations were performed on 10 subjects not included in the optimization database.

## 3 Results

**FIGURE 3:**
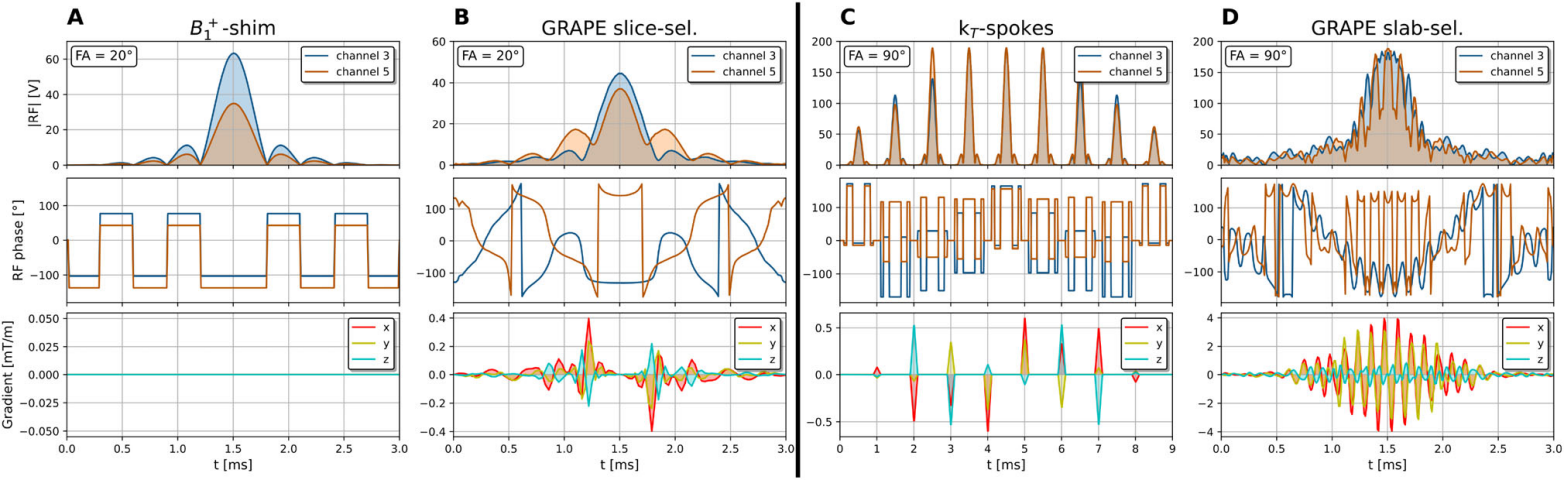
Representation of the different optimized pulse shapes. (A) $B_{1}$‐shimmed sinc‐shaped radiofrequency pulse (without gradients). (B) GRAPE pulse optimized for slice‐selective excitation. (C) k
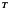
‐spokes pulse for slab‐selective excitation. (D) GRAPE pulse optimized for slab‐selective excitation.

The RF and gradient waveforms of the $B_{1}^{+}$‐shimmed sinc pulse, slice‐selective GRAPE (optimized for the whole brain, that is, Strategy 1), k
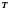
‐spokes, and slab‐selective GRAPE pulses are shown in Figure 3. While the slice‐selective GRAPE pulse maintains a sinc‐like RF waveform, it is distinguished from the $B_{1}^{+}$‐shimmed pulse by altered side‐lobe scaling, slight phase variations, and the presence of low‐amplitude gradients. In contrast, the RF shape of the slab‐selective GRAPE pulse deviates from a sinc shape by fast oscillations, which are also present in the gradients fields of relatively high amplitudes. Finally, the GRAPE pulse is three times shorter than the k
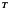
‐spokes pulse, while depositing only half of the energy (1741 and 3405 mJ, respectively).

### 3.1 Slice‐Selection

**FIGURE 4:**
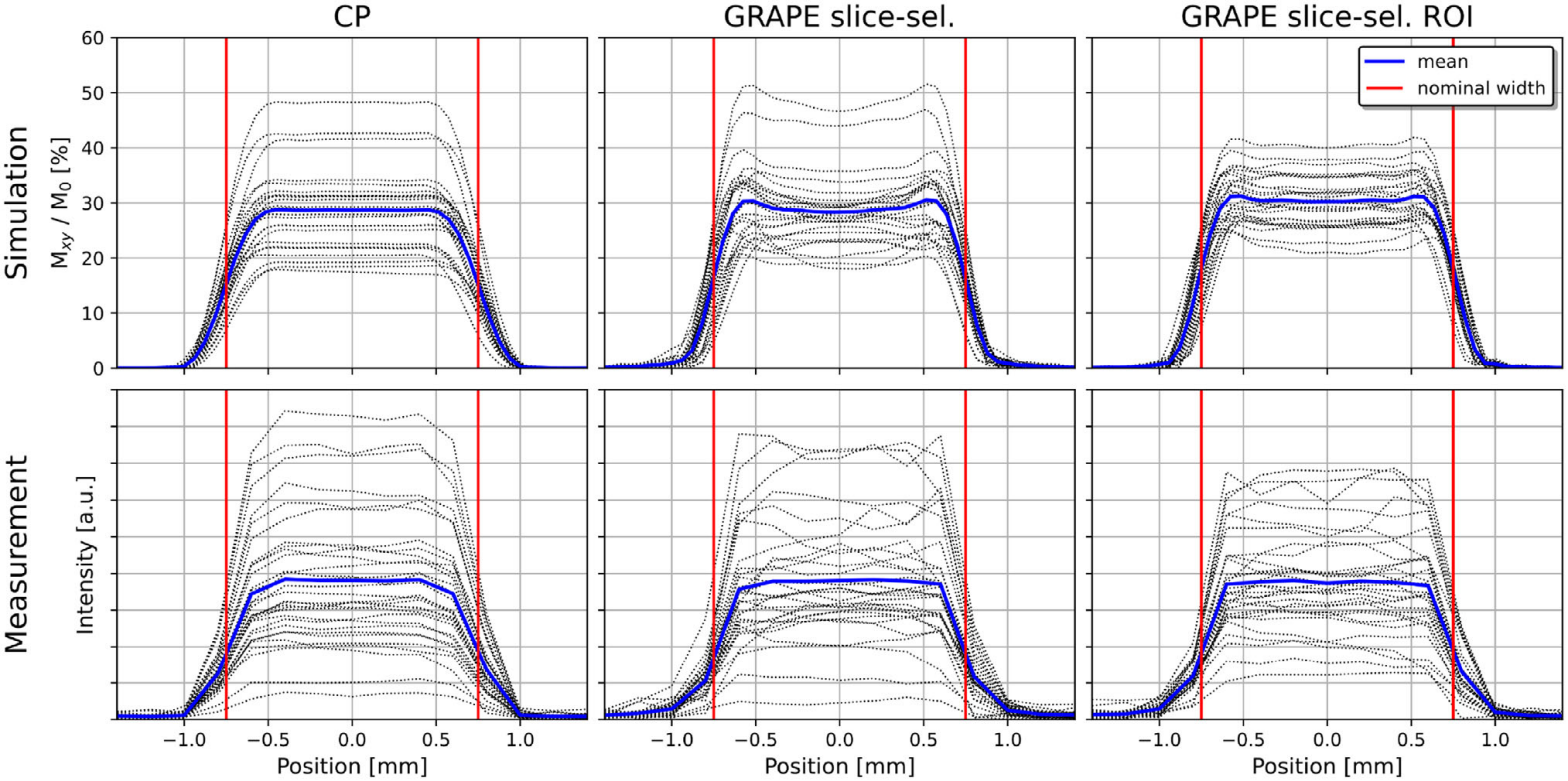
Simulated and measured slice profiles: The circularly polarized (CP) pulse (left), the GRAPE pulse for slice‐selective excitation optimized on the whole brain (middle) and a set of GRAPE slice‐selective pulses, each optimized for a specific region of interest (ROI) around the slice position (right). Dotted lines represent slice profiles at various brain locations (distributed equidistantly on a 4 cm grid). The blue line indicates the mean slice profile and red lines denote the nominal slice width (1.5 mm). Simulation results show the transverse magnetization $M_{xy}$, normalized to the equilibrium magnetization $M_{0}$.

Figure 4 presents the slice profiles generated by the slice‐selective pulses, showing simulation and measurement results from brain voxels arranged on a 4 cm grid (dotted black lines). Measurements were not conducted for the $B_{1}^{+}$‐shimmed pulses, as their slice profiles were presumed to be almost identical to those of the CP pulse. All pulses exhibited very similar slice profiles.

**FIGURE 5:**
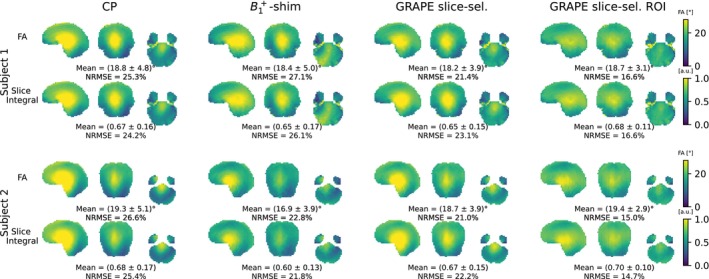
Bloch simulation results for slice‐selective RF pulses. Columns correspond to circularly polarized (CP), $B_{1}^{+}$‐shimmed, and GRAPE‐optimized pulses. GRAPE pulses were optimized either on the whole brain or for the specific slice location (region of interest, ROI). For each case, the flip angle (FA) in the center of the respective slice and the integral over the slice in slice‐selection direction (“slice integral”) were determined.

Bloch simulation results, depicted in Figure 5, show the FA at the center of each slice (upper row) and the integral of the transverse magnetization in the slice‐selection direction (lower row) for two subjects. While the FA and slice integral generally show very good agreement, the GRAPE pulse optimized on the whole brain exhibits slightly reduced homogeneity in its slice integral. This diminished homogeneity is attributed to phase deviations along the slice‐selection direction, which diminish the effective signal in some voxels.

The $B_{1}^{+}$‐shimmed pulse slightly improves signal in the cerebellum, but in subject 1 the homogeneity is worse than for the CP pulse and in subject two the mean FA drops significantly, while depositing more RF energy (101 mJ vs. 87 mJ for CP). Slice‐selective GRAPE pulses optimized on the whole brain achieve improved homogeneity while maintaining similar RF energy (87.7 mJ). GRAPE slice‐selective ROI pulses further enhance homogeneity and even reduce RF energy (78.4 mJ averaged over all slices).

**FIGURE 6:**
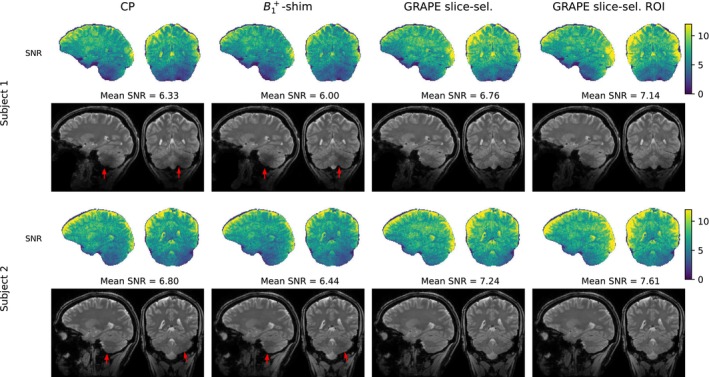
GRE images and corresponding SNR maps acquired from two subjects are depicted employing either circularly polarized (CP), $B_{1}^{+}$‐shimmed or GRAPE pulses (either optimized on the whole brain or for the specific region of interest (ROI) of the respective slices). Red arrows highlight areas with reduced signal in the cerebellum.

Measurements with slice‐selective GRAPE pulses are in good agreement with the simulation results (cf. Figure 6). GRAPE pulses consistently yielded a higher mean signal‐to‐noise ratio than both CP and $B_{1}^{+}$‐shimmed pulses. Moreover, the signal reduction typically observed in the cerebellum was mitigated through the use of GRAPE pulses.

### 3.2 Slab‐Selection

**FIGURE 7:**
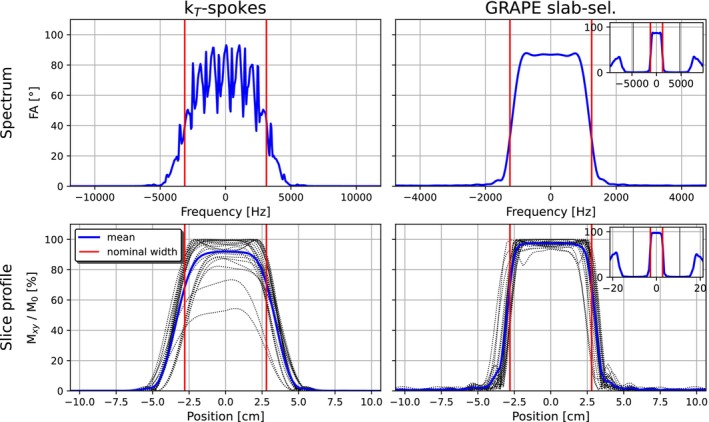
Simulated spectral responses and slab profiles obtained from Bloch simulations are displayed for k
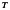
‐spokes and slab‐selective GRAPE pulses. Slab profiles represent the transverse magnetization $M_{xy}$, normalized to the initial magnetization $M_{0}$. Dotted lines denote slab profiles corresponding to different slab‐center positions, varied in 4 cm increments across the whole brain. Red lines denote the nominal bandwidth or simulated slab thickness. For the GRAPE pulse, a zoomed‐out view of the spectral response and the slab profile is shown in the upper right corner.

Simulations were performed to assess the spectral responses and slab profiles of the slab‐selective pulses (cf. Figure 7). While the mean slab profiles are close to the target profile, the spectrum of the k
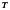
‐spokes pulse exhibited oscillations superimposed on its anticipated envelope.

The spectrum of the GRAPE pulse exhibited side lobes at large frequencies (cf. small plot in the upper right corner of the GRAPE spectrum in Figure 7). Because the frequency offsets were drawn from a uniform distribution on a limited interval, the flip angle at higher offsets was not taken into account during the optimization process.

**FIGURE 8:**
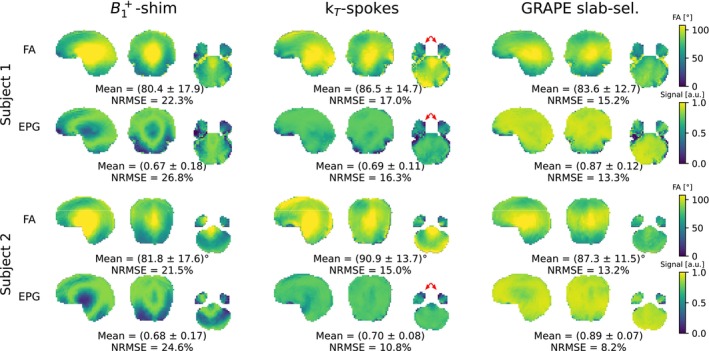
Flip angle maps from Bloch simulations and corresponding signal maps from extended phase graph (EPG) simulations are shown for $B_{1}^{+}$‐shimmed, k
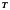
‐spokes and GRAPE pulses. The EPG simulation was performed for the central echo in a slab‐selective variable flip angle turbo spin echo (TSE) sequence. Slab‐selection gradients were omitted to enable simulations of the whole brain. The first echo spacing for $B_{1}^{+}$‐shimmed and k
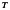
‐spokes pulses was 19.7 ms and for GRAPE pulses 12.5 ms, leading to increased signal. Red arrows highlight regions of pronounced signal loss in the temporal lobes.

Results from Bloch and extended phase graph (EPG) simulations utilizing $B_{1}^{+}$‐shimmed, k
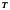
‐spokes and slab‐selective GRAPE pulses are shown in Figure 8. These simulations were performed without slab‐selection gradients to estimate the performance of the pulses independent for the entire brain. GRAPE pulses achieved more homogeneous FAs and signal as well as significantly increased overall signal at the central echo of the EPG (which is attributed to a shorter first echo spacing of 12.5 ms compared to 19.7 ms with $B_{1}^{+}$‐shimmed and k
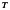
‐spokes pulses).

$B_{1}^{+}$‐shimmed pulses exhibited insufficient homogeneity, leading to substantial signal loss in the cerebellum due to low FAs as well as signal loss in the central brain region due to high FAs. In contrast, k
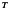
‐spokes pulses improved homogeneity but suffered from signal loss in the anterior parts of the frontal lobes, where strong off‐resonances occur.

**FIGURE 9:**
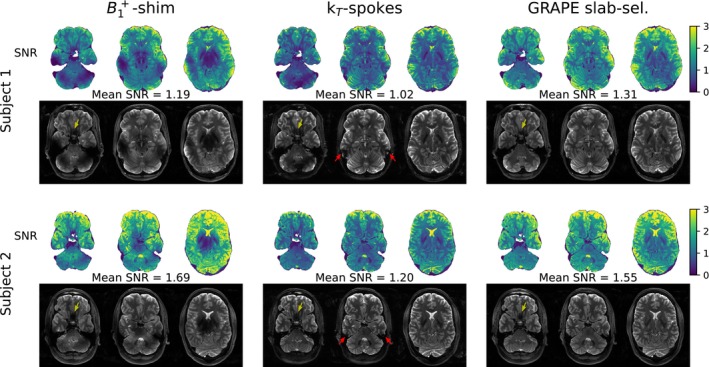
Images of a slab‐selective variable flip angle turbo spin echo sequence and corresponding SNR maps are shown for two subjects employing either $B_{1}^{+}$‐shimmed, k
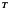
‐spokes or GRAPE slab‐selective pulses for excitation. Yellow and red arrow indicate signal loss in the frontal and temporal lobes, respectively.

Three slices of the measured slab are displayed in Figure 9. $B_{1}^{+}$‐shimmed pulses resulted in significant signal loss in the cerebellum, the temporal lobes and the center of the brain. The GRAPE pulse resulted in a higher total SNR compared to k
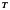
‐spokes pulses, consistent with EPG simulation predictions. While all pulses experienced signal loss in the frontal lobe, GRAPE pulses mitigated signal loss in the temporal lobes.

Table 1 summarizes the quantitative values obtained from simulations and measurements of all slice‐ and slab‐selective pulses. Flip angles, slice integrals, EPGs and SNR were averaged over 10 simulated subjects, while SNR values were averaged over both measured subjects. The slice‐selective pulses apply the same energy for each slice, only the GRAPE ROI pulses were optimized for each slice separately and therefore the averaged energy over all slices is given.

**TABLE 1:** Summary of quantitative simulation and measurement results of all pulses. Flip angles and slice integrals/EPGs were averaged over 10 simulated subjects. Simulations of the $B_{1}^{+}$‐shimmed pulses and all SNR values were averaged over both measured subjects.

Pulse		Duration (ms)	Energy (mJ)	FA (°)	Slice integral/EPG (a.u.)	SNR
Slice‐selective	CP	3	87.0	19.5±5.5	0.69±0.18	6.56
	B 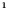 ^+^‐shim	3	101.0	19.2±4.5	0.63±0.15	6.22
	GRAPE	3	87.7	19.1±4.1	0.67±0.15	7.00
	GRAPE ROI	3	78.4^a^	19.8±3.0	0.71±0.11	7.38
Slab‐selective	B 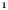 ^+^‐shim	10.5	1627	81.1±17.8	0.68±0.18	1.44
	k 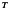 ‐spokes	9	3405	91.1±13.6	0.69±0.08	1.11
	GRAPE	3	1741	87.9±11.8	0.88±0.08	1.43

^a^ The energy was averaged over all slices.

To confirm adaptability of the pulses to different FOV orientations, slice‐selective and slab‐selective GRAPE pulses (both optimized over the whole brain) were acquired in all main directions (cf. Figures S1 and S2).

## 4 Discussion

We propose a method to design GRAPE pulses with a specified spectral response and applied these pulses for spatially‐selective excitation in a 2D GRE and a slab‐selective TSE sequence. In general, GRAPE pulses increased overall SNR and significantly reduced signal loss in regions affected by low transmit field sensitivities or high off‐resonances. Furthermore, it was shown that spatially‐selective GRAPE pulses can be designed as UPs [20], eliminating the need for additional subject‐specific calibration during the imaging session. Figure S3 shows robust performance of spatially‐selective GRAPE UPs in 10 subjects confirming the applicability of UPs for our pulse design method. A comparison to subject‐tailored (ST) GRAPE pulses is shown in Figures S4 and S5. ST pulses could improve the performance of GRAPE pulses even further, but require extensive calculation time, rendering them unfeasible for clinical applications. However, a combination of pre‐optimized pulses and a quick subject‐specific adjustment as proposed with standardized UPs [46] or fast online‐customized (FOCUS) pulses [47] could offer a practical compromise.

In the slice‐selective experiments, the GRAPE pulse optimized for the whole brain achieved signal homogeneity comparable to that of $B_{1}^{+}$‐shimmed pulse in simulations, but higher measured SNR. This resulted in a visible increase in signal intensity in the cerebellum. Further enhancements in homogeneity and overall signal were achieved using GRAPE pulses optimized for the specific slice position. These position‐specific pulses were also compatible with the concept of UP. Flexibility was only moderately constrained with respect to large rotations, as the weighting term employed in the pulse design ensures robustness against small shifts and tilts of the FOV [31]. Therefore, the GRAPE pulses optimized for the whole brain provide slightly more flexibility with regard to FOV shifts and rotations, but at the expense of FA homogeneity and more pulse energy (12% more than GRAPE ROI pulses. cf. Table 1).

The slab‐selective GRAPE pulse optimized for the whole brain achieved substantially improved flip angle homogeneity, with a normalized root mean square error (NRMSE) averaged over 10 subjects of 13.4%, compared to 21.5% for the slice‐selective GRAPE pulse and 15.2% for the ROI‐specific optimized slice‐selective GRAPE pulses. We assume that the fast oscillations in the RF and gradient shapes of the slab‐selective GRAPE pulse were the primary cause for the improved homogeneity. These oscillations also lead to side lobes outside the frequency interval on which the pulse was optimized (cf. Figure 7). In case of slab excitation, such side lobes far from the main peak do not generate significant interfering signal, since the signal is far enough from the imaging field of view, where both the transmit and receive sensitivities are negligible. In contrast, for slice‐selective applications, similar side lobes would excite magnetization within the brain leading to undesirable signal. Therefore, side lobes were suppressed for slice‐selective GRAPE pulses during the optimization by drawing frequency offsets from a wider Gaussian distribution.

The universal k
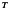
‐spokes pulse did not achieve flip angle homogeneity comparable to the slab‐selective GRAPE pulse, yielding a NRMSE of 14.9%. Furthermore, the GRAPE pulses produced a signal gain of approximately 28% in EPG simulations compared to the k
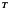
‐spokes pulse. The reduced pulse duration led to a shorter echo spacing between the excitation and the 180° refocusing pulse (19.7 ms with k
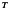
‐spokes and 12.5 ms with the GRAPE pulse), thereby increasing the overall signal. The measured SNR maps also showed a gain of the signal by over 29% in both subjects. Moreover, a slight reduction of the SNR in the center of the brain (at the upper boundary of the slab) with the GRAPE excitation pulse was observable in both subjects. This reduction can be most likely explained by greater phase variations introduced by the GRAPE pulse at off‐center frequencies (mapped to the borders of the slab when a slab‐selection gradient is present), which reduced the coherence of the echo train and thus the resulting signal. A stronger weighting of the phase linearity (with an increased parameter $\lambda_{\text{ph}}$) might improve the signal at the slab boundaries in TSE applications, where satisfying the CPMG condition is essential.

Further reduction of the pulse duration could potentially eliminate the need for the 180° refocusing pulse altogether. This might enhance SNR further, as the echo train length could be shortened even more and by avoiding signal loss due to imperfect refocusing (as explained in detail by Snyder et al. [48]).

Another advantage of the GRAPE slab‐selective pulse over the k
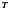
‐spokes pulse is its robustness to off‐resonances. As illustrated in Figure 7, the k
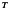
‐spokes pulse exhibits a spectrum, which is sensitive to off‐resonances leading to signal dropouts in certain brain areas. This phenomenon is attributed to the fact that the k
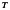
‐spokes pulse is composed of nine sub‐pulses, which induce some kind of “binomial effect”. At each sub‐pulse the gradient polarity is reversed, but the off‐resonances due to static field deviations remain constant. Phase differences from gradient fields are rewound at the center of each sub‐pulse, whereas phase differences originating from off‐resonances accumulate across successive sub‐pulses. This cumulative effect results in slice profiles that vary across voxels depending on the magnitude of the $B_{0}$ shifts, rather than a direct translation of the spectrum to a corresponding slice profile. In contrast, the GRAPE pulse's mean spectrum, in the presence of gradient fields, was translated to a mean slice profile with minimal changes, because the gradient polarity was not switching. Although each sub‐pulse of the k
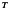
‐spokes pulse has a nominal bandwidth of 6.25 kHz, from which one would assume robustness to off‐resonances, the binomial effect causes pronounced signal dropouts, particularly in the temporal lobes. In contrast, the GRAPE pulse has an actual bandwidth of about 2.5 kHz and demonstrates greater robustness to static field variations, thereby preserving signal in the temporal lobes. Moreover, the GRAPE pulse slightly mitigates signal dropouts in the frontal lobe compared to the k
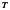
‐spokes pulse as well as the $B_{1}^{+}$‐shimmed pulse (cf. Figure 9).

When large head motion occurs the pulse performance of GRAPE pulses could be affected compared to conventional CP pulses, but the effect is negligible compared to imaging artifacts. Especially the design of UPs creates robustness against variations in the head position, because the database includes subjects with different head positions.

In this work, we have demonstrated two applications benefiting from spatially‐selective GRAPE pulses. However, their usage is not limited to these examples and can be extended to other sequences. For example, GRAPE pulses can also be employed for spatially‐selective refocusing. In preliminary work, we demonstrated the application of 90° excitation and 180° refocusing with slice‐selective GRAPE pulses in a spin echo sequence in combination with simultaneous multi‐slice acquisition (SMS) [49].

Particularly, for other slab‐selective applications these pulses could be of interest. The TSE sequence used in our work poses a considerable challenge due to the strict requirement to satisfy the CPMG condition. In contrast, other slab‐selective sequences, less sensitive to phase variations, can benefit even more from a GRAPE excitation pulse. For example, we have achieved promising preliminary results using slab‐selective GRAPE pulses for whole‐brain MRSI [50].

Another potential field of application is spectral‐selection. The pulses are basically optimized for a specific spectral response and employed for spatial‐selection by applying gradient fields. However, without gradient fields, the pulses can be used to excite magnetization only in a certain spectral range, for example, for fat or water suppression.

## 5 Conclusions

In this work, we present a method to design universal GRAPE pulses with a specified spectral response. The proposed approach is demonstrated in a 2D GRE and a slab‐selective TSE sequence. In both applications, universal GRAPE pulses result in substantially increased SNR in two subjects not included in the optimization database. Additionally, the method exhibits improved robustness to static field deviations compared to k
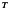
‐spokes pTx pulses. These findings pave the way for novel applications exploiting the full potential of pTx.

## Funding

This work was supported by the European Union Horizon 2020 Research and Innovation program, Grant agreement 885876 (AROMA).

## Supporting information




**Figure S1.** Slice‐selective GRAPE pulses were tested in sagittal, coronal, transversal, and a 45° diagonal orientation within a 2D multi‐slice GRE sequence (102 slices respectively) to confirm the adaptability across all main orientations. The images were reformatted to sagittal and cor‐onal orientations for display.
**Figure S2.** Acquisition of all main directions with a slab‐selective GRAPE excitation pulse in a 3D TSE sequence.
**Figure S3.** Boxplot illustrating inter‐subject variability of different slice‐ (left) and slab‐selective (right) pulses in Bloch simulations. Upper plots show the distribution of the mean FA across 10 subjects and lower plots show the FA‐NRMSE. GRAPE pulses were designed as universal pulses (GRAPE and GRAPE ROI) or subject‐tailored (ST) pulses (GRAPE ST and GRAPE ROI ST).
**Figure S4.** Bloch simulation results for slice‐selective RF pulses. GRAPE pulses were optimized either on the whole brain (first two columns) or for the specific slice location (last two columns). Columns 1 and 3 show UPs. Columns 2 and 4 show subject‐tailored (ST) pulses. For each case, the flip angle (FA) in the center of the respective slice and the integral over the slice in slice‐selection direction (“slice integral”) were determined.
**Figure S5.** Flip angle maps from Bloch simulations and corresponding signal maps from extended phase graph (EPG) simulations are shown for kT‐spokes and GRAPE pulses (UP in the second column and subject‐tailored (ST) in the third column).

## Data Availability

Research data are not shared.

## References

[1] M. Collins Christopher and B. Smith Michael , “Signal‐To‐Noise Ratio and Absorbed Power as Functions of Main Magnetic Field Strength, and Definition of “$90^{\circ }$” RF Pulse for the Head in the Birdcage Coil,” Magnetic Resonance in Medicine 45, no. 4 (2001): 684–691. 11283997 10.1002/mrm.1091

[2] R. Pohmann , O. Speck , and K. Scheffler , “Signal‐To‐Noise Ratio and MR Tissue Parameters in Human Brain Imaging at 3, 7, and 9.4 Tesla Using Current Receive Coil Arrays,” Magnetic Resonance in Medicine 75, no. 2 (2016): 801–809. 25820458 10.1002/mrm.25677

[3] M. E. Ladd , B. Peter , M. Martin , et al., “Pros and Cons of Ultra‐High‐Field MRI/MRS for Human Application,” Progress in Nuclear Magnetic Resonance Spectroscopy 109 (2018): 1–50. 30527132 10.1016/j.pnmrs.2018.06.001

[4] Ö. Can , W. Matthew , E. Wendy , et al., “Use of a Commercial 7‐T MRI Scanner for Clinical Brain Imaging: Indications, Protocols, Challenges, and Solutions—A Single‐Center Experience,” American Journal of Roentgenology 221, no. 6 (2023): 788–804. 37377363 10.2214/AJR.23.29342 PMC10825876

[5] T. S. Ibrahim , R. Lee , A. M. Abduljalil , B. A. Baertlein , and P. L. Robitaille , “Dielectric Resonances and B1 Field Inhomogeneity in UHFMRI: Computational Analysis and Experimental Findings,” Magnetic Resonance Imaging 19, no. 2 (2001): 219–226. 11358660 10.1016/s0730-725x(01)00300-9

[6] A. G. Webb and C. M. Collins , “Parallel Transmit and Receive Technology in High‐Field Magnetic Resonance Neuroimaging,” International Journal of Imaging Systems and Technology 20, no. 1 (2010): 2–13.

[7] T. S. Ibrahim , L. Robert , B. A. Baertlein , K. Allahyar , and P.‐M. L. Robitaille , “Application of Finite Difference Time Domain Method for the Design of Birdcage RF Head Coils Using Multi‐Port Excitations,” Magnetic Resonance Imaging 18, no. 6 (2000): 733–742. 10930783 10.1016/s0730-725x(00)00143-0

[8] U. Katscher , P. Börnert , C. Leussler , and J. S. Van den Brink , “Transmit SENSE,” Magnetic Resonance in Medicine 49, no. 1 (2003): 144–150. 12509830 10.1002/mrm.10353

[9] P. Francesco , B. Arian , J. V. Hajnal , and S. J. Malik , “Parallel Transmission for Ultrahigh‐Field Imaging,” NMR in Biomedicine 29, no. 9 (2016): 1145–1161. 25989904 10.1002/nbm.3313 PMC4995736

[10] N. Williams Sydney and M. E. Paul , “Gunamony Shajan. Ultra‐High Field MRI: Parallel‐Transmit Arrays and RF Pulse Design,” Physics in Medicine & Biology 68, no. 2 (2023): 02TR02. 10.1088/1361-6560/aca4b7 36410046

[11] M. Weihua , M. B. Smith , and C. M. Collins , “Exploring the Limits of RF Shimming for High‐Field MRI of the Human Head,” Magnetic Resonance in Medicine 56, no. 4 (2006): 918–922. 16958070 10.1002/mrm.21013 PMC4040521

[12] P. John , N. Dwight , and M. Albert , “A k‐Space Analysis of Small‐Tip‐Angle Excitation,” Journal of Magnetic Resonance 81 (1989): 43–56.

[13] M. A. Cloos , N. Boulant , M. Luong , et al., “K T‐Points: Short Three‐Dimensional Tailored RF Pulses for Flip‐Angle Homogenization Over an Extended Volume,” Magnetic Resonance in Medicine 67, no. 1 (2012): 72–80. 21590724 10.1002/mrm.22978

[14] S. Suwit , Y. Yip Chun , D. C. Noll , F. E. Boada , and S. V. Andrew , “Fast‐Kz Three‐Dimensional Tailored Radiofrequency Pulse for Reduced B1 Inhomogeneity,” Magnetic Resonance in Medicine 55, no. 4 (2006): 719–724. 16526012 10.1002/mrm.20840 PMC3076290

[15] S. Kawin , L. L. Wald , A. Vijayanand , et al., “Parallel RF Transmission With Eight Channels at 3 Tesla,” Magnetic Resonance in Medicine 56, no. 5 (2006): 1163–1171. 17036289 10.1002/mrm.21042

[16] G. Saïb , V. Gras , F. Mauconduit , et al., “kT‐Spokes Combining kT‐Points With Spokes to Ease Ramp Pulse Design for TOF Slab Selection With Parallel Transmission at 7T,” in Joint Annual Meeting ISMRM‐ESMRMB (ISMRM, 2018).

[17] R. Jamil , F. Mauconduit , V. Gras , and N. Boulant , “General Gradient Delay Correction Method in Bipolar Multispoke RF Pulses Using Trim Blips,” Magnetic Resonance in Medicine 85, no. 2 (2021): 1004–1012. 32851654 10.1002/mrm.28478

[18] N. Khaneja , T. Reiss , C. Kehlet , T. Schulte‐Herbrüggen , and S. J. Glaser , “Optimal Control of Coupled Spin Dynamics: Design of NMR Pulse Sequences by Gradient Ascent Algorithms,” Journal of Magnetic Resonance 172, no. 2 (2005): 296–305. 15649756 10.1016/j.jmr.2004.11.004

[19] P. De Fouquieres , S. G. Schirmer , S. J. Glaser , and K. Ilya , “Second order Gradient Ascent Pulse Engineering,” Journal of Magnetic Resonance 212, no. 2 (2011): 412–417. 21885306 10.1016/j.jmr.2011.07.023

[20] V. Gras , A. Vignaud , A. Amadon , D. Le Bihan , and N. Boulant , “Universal Pulses: A New Concept for Calibration‐Free Parallel Transmission,” Magnetic Resonance in Medicine 77, no. 2 (2017): 635–643. 26888654 10.1002/mrm.26148

[21] V. Gras , F. Mauconduit , A. Vignaud , et al., “Design of Universal Parallel‐Transmit Refocusing k T ‐Point Pulses and Application to 3D T 2 ‐Weighted Imaging at 7T,” Magnetic Resonance in Medicine 80, no. 1 (2018): 53–65. 29193250 10.1002/mrm.27001

[22] M. Ronald , A. Clark Ian , A. Maguire Eleanor , et al., “Universal Pulses for Homogeneous Excitation Using Single Channel Coils,” Magnetic Resonance Imaging 92, no. July (2022): 180–186. 35820546 10.1016/j.mri.2022.07.002

[23] L. Van Damme , F. Mauconduit , T. Chambrion , N. Boulant , and V. Gras , “Universal Nonselective Excitation and Refocusing Pulses With Improved Robustness to Off‐Resonance for Magnetic Resonance Imaging at 7 Tesla With Parallel Transmission,” Magnetic Resonance in Medicine 85, no. 2 (2021): 678–693. 32755064 10.1002/mrm.28441

[24] V. Gras , F. Mauconduit , A. Vignaud , et al., “PASTeUR: Package of Anatomical Sequences Using Parallel Transmission UniveRsal kT‐Point Pulses,” in Joint Annual Meeting ISMRM‐ESMRMB (ISMRM, 2019).

[25] M. Zhang , N. Arango , Y. Arefeen , et al., “Stochastic‐Offset‐Enhanced Restricted Slice Excitation and $180^{\circ }$ Refocusing Designs With Spatially Non‐Linear ΔB0 Shim Array Fields,” Magnetic Resonance in Medicine 90, no. 6 (2023): 2572–2591. 37667645 10.1002/mrm.29827 PMC10699120

[26] M. A. Bernstein , K. K. Franklin , and Z. Z. Joe , Handbook of MRI Pulse Sequences (Elsevier Academic Press, 2004).

[27] P. Virtanen , R. Gommers , T. E. Oliphant , et al., “SciPy 1.0: Fundamental Algorithms for Scientific Computing in Python,” Nature Methods 17, no. 3 (2020): 261–272. 32015543 10.1038/s41592-019-0686-2 PMC7056644

[28] D. Loewen , E. D. Pracht , V. Gras , et al., “Design of Calibration‐Free RF Pulses for T2‐Weighted Single‐Slab 3D Turbo‐Spin‐Echo Sequences at 7T Utilizing Parallel Transmission,” Magnetic Resonance in Medicine 92 (2024): 2037–2050. 39054786 10.1002/mrm.30212

[29] S. Kanayamay , S. Kuhara , and K. Satoh , “In Vivo Rapid Magnetic Field Measurement and Shimming Using Single Scan Differential Phase Mapping,” Magnetic Resonance in Medicine 36, no. 4 (1996): 637–642. 8892219 10.1002/mrm.1910360421

[30] V. L. Yarnykh , “Actual Flip‐Angle Imaging in the Pulsed Steady State: A Method for Rapid Three‐Dimensional Mapping of the Transmitted Radiofrequency Field,” Magnetic Resonance in Medicine 57, no. 1 (2007): 192–200. 17191242 10.1002/mrm.21120

[31] V. Gras , B. A. Poser , X. Wu , R. Tomi‐Tricot , and N. Boulant , “Optimizing BOLD Sensitivity in the 7T Human Connectome Project Resting‐State fMRI Protocol Using Plug‐And‐Play Parallel Transmission,” NeuroImage 195 (2019): 1–10. 30923027 10.1016/j.neuroimage.2019.03.040

[32] X. Wu , E. J. Auerbach , A. T. Vu , et al., “High‐Resolution Whole‐Brain Diffusion MRI at 7T Using Radiofrequency Parallel Transmission,” Magnetic Resonance in Medicine 80, no. 5 (2018): 1857–1870. 29603381 10.1002/mrm.27189 PMC6107381

[33] J. P. Mugler , S. Bao , R. V. Mulkern , et al., “Optimized Single‐Slab Three‐Dimensional Spin‐Echo MR Imaging of the Brain,” Radiology 216, no. 3 (2000): 891–899. 10966728 10.1148/radiology.216.3.r00au46891

[34] J. P. Mugler , “Optimized Three‐Dimensional Fast‐Spin‐Echo MRI,” Journal of Magnetic Resonance Imaging 39, no. 4 (2014): 745–767. 24399498 10.1002/jmri.24542

[35] J. P. Mugler , B. Kiefer , and J. R. Brookeman , Three‐Dimensional T2‐Weighted Imaging of the Brain Using Very Long Spin‐Echo Trains (ISMRM, 2000).

[36] J. Hennig , M. Weigel , and K. Scheffler , “Calculation of Flip Angles for Echo Trains With Predefined Amplitudes With the Extended Phase Graph (EPG)‐algorithm: Principles and Applications to Hyperecho and TRAPS Sequences,” Magnetic Resonance in Medicine 51, no. 1 (2004): 68–80. 14705047 10.1002/mrm.10658

[37] E. D. Pracht , T. Feiweier , P. Ehses , et al., “SAR and Scan‐Time Optimized 3D Whole‐Brain Double Inversion Recovery Imaging at 7T,” Magnetic Resonance in Medicine 79, no. 5 (2018): 2620–2628. 28905416 10.1002/mrm.26913

[38] D. Mitsouras , R. V. Mulkern , and F. J. Rybicki , “Strategies for Inner Volume 3D Fast Spin Echo Magnetic Resonance Imaging Using Nonselective Refocusing Radio Frequency Pulses,” Medical Physics 33, no. 1 (2006): 173–186. 16485424 10.1118/1.2148331 PMC1414094

[39] H. Y. Carr and E. M. Purcell , “Effects of Diffusion on Free Precession in Nuclear Magnetic Resonance Experiments,” Physics Review 94, no. 3 (1954): 630–638.

[40] S. Meiboom and D. Gill , “Modified Spin‐Echo Method for Measuring Nuclear Relaxation Times,” Review of Scientific Instruments 29, no. 8 (1958): 688–691.

[41] M. Uecker , P. Lai , M. J. Murphy , et al., “ESPIRiT—An Eigenvalue Approach to Autocalibrating Parallel MRI: Where SENSE Meets GRAPPA,” Magnetic Resonance in Medicine 71, no. 3 (2014): 990–1001. 23649942 10.1002/mrm.24751 PMC4142121

[42] M. Blumenthal , M. Heide , C. Holme , et al., “mrirecon/bart: Version 0.9.00,” 2023.

[43] P. M. Robson , A. K. Grant , A. J. Madhuranthakam , R. Lattanzi , D. K. Sodickson , and C. A. McKenzie , “Comprehensive Quantification of Signal‐To‐Noise Ratio and *g*‐Factor for Image‐Based and *k* ‐Space‐Based Parallel Imaging Reconstructions,” Magnetic Resonance in Medicine 60, no. 4 (2008): 895–907. 18816810 10.1002/mrm.21728 PMC2838249

[44] E. Philipp , B. Daniel , S. Rüdiger , E. D. Pracht , and S. Tony , “Whole‐Brain B1‐Mapping Using Three‐Dimensional DREAM,” Magnetic Resonance in Medicine 82, no. 3 (2019): 924–934. 31038244 10.1002/mrm.27773

[45] F. A. Breuer , M. Blaimer , M. F. Mueller , et al., “Controlled Aliasing in Volumetric Parallel Imaging (2D CAIPIRINHA),” Magnetic Resonance in Medicine 55, no. 3 (2006): 549–556. 16408271 10.1002/mrm.20787

[46] L. S. Caroline , M. Franck , M. Aurélien , B. Nicolas , and G. Vincent , “Standardized Universal Pulse: A Fast RF Calibration Approach to Improve Flip Angle Accuracy in Parallel Transmission,” Magnetic Resonance in Medicine 87, no. 6 (2022): 2839–2850. 35122302 10.1002/mrm.29180

[47] H. Jürgen , L. Patrick , G. Rene , et al., “Fast Online‐Customized (FOCUS) Parallel Transmission Pulses: A Combination of Universal Pulses and Individual Optimization,” Magnetic Resonance in Medicine 85, no. 6 (2021): 3140–3153. 33400302 10.1002/mrm.28643

[48] J. Snyder , P. Seres , and A. H. Wilman , “SNR Penalties From Loss of Stimulated Echoes When Using Slab Selective Excitation in 3D Fast Spin Echo Imaging With Long Echo Trains,” NMR in Biomedicine 36, no. 5 (2022): e4881. 36427186 10.1002/nbm.4881

[49] M. Veldmann , D. Loewen , and T. Stoecker , Improving Signal Homogeneity of Whole‐Brain DWI at 7T With Universal Multiband GRAPE pTx Pulses (ISMRM, 2025).

[50] Y. Voelzke , D. Loewen , E. D. Pracht , et al., GRAPE‐Based Slab‐Selective Universal Parallel Transmit Excitation Pulses for Whole‐Brain MRSI at 7 Tesla (ISMRM, 2025).

