# Supplementary material for: Calibration‐Free GRAPE pTx Pulses for Homogeneous Spatial‐Selective Excitation at 7T
Source: Magn Reson Med. 2026 Jan 26;95(6):3104–15. doi: 10.1002/mrm.70266 (PMC13049268; doi:10.1002/mrm.70266)
Supplement: Supplementary file 1 — Figure S1. Slice‐selective GRAPE pulses were tested in sagittal, coronal, transversal, and a 45° diagonal orientation within a 2D multi‐slice GRE sequence (102 slices respectively) to confirm the adaptability across all main orientations. The images were reformatted to sagittal and cor‐onal orientations for display. Figure S2. Acquisition of all main directions with a slab‐selective GRAPE excitation pulse in a 3D TSE sequence. Figure S3. Boxplot illustrating inter‐subject variability of different slice‐ (left) and slab‐selective (right) pulses in Bloch simulations. Upper plots show the distribution of the mean FA across 10 subjects and lower plots show the FA‐NRMSE. GRAPE pulses were designed as universal pulses (GRAPE and GRAPE ROI) or subject‐tailored (ST) pulses (GRAPE ST and GRAPE ROI ST). Figure S4. Bloch simulation results for slice‐selective RF pulses. GRAPE pulses were optimized either on the whole brain (first two columns) or for the specific slice location (last two columns). Columns 1 and 3 show UPs. Columns 2 and 4 show subject‐tailored (ST) pulses. For each case, the flip angle (FA) in the center of the respective slice and the integral over the slice in slice‐selection direction (“slice integral”) were determined. Figure S5. Flip angle maps from Bloch simulations and corresponding signal maps from extended phase graph (EPG) simulations are shown for kT‐spokes and GRAPE pulses (UP in the second column and subject‐tailored (ST) in the third column). [file MRM-95-3104-s001.docx]

Figure S1: Slice-selective GRAPE pulses were tested in sagittal, coronal, transversal, and a 45° diagonal orientation within a 2D multi-slice GRE sequence (102 slices respectively) to confirm the adaptability across all main orientations. The images were reformatted to sagittal and cor-onal orientations for display.


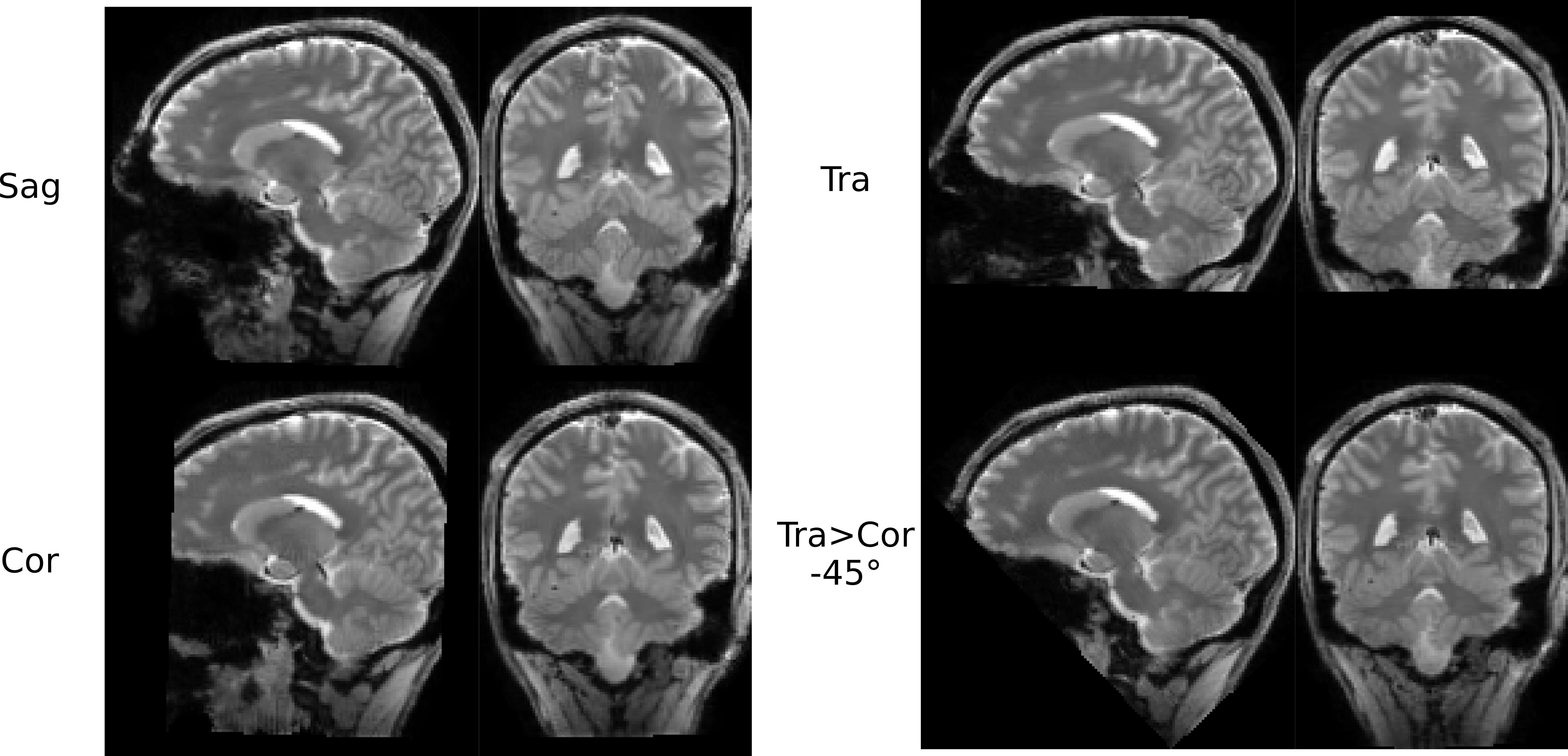


Figure S2: Acquisition of all main directions with a slab-selective GRAPE excitation pulse in a 3D TSE sequence.
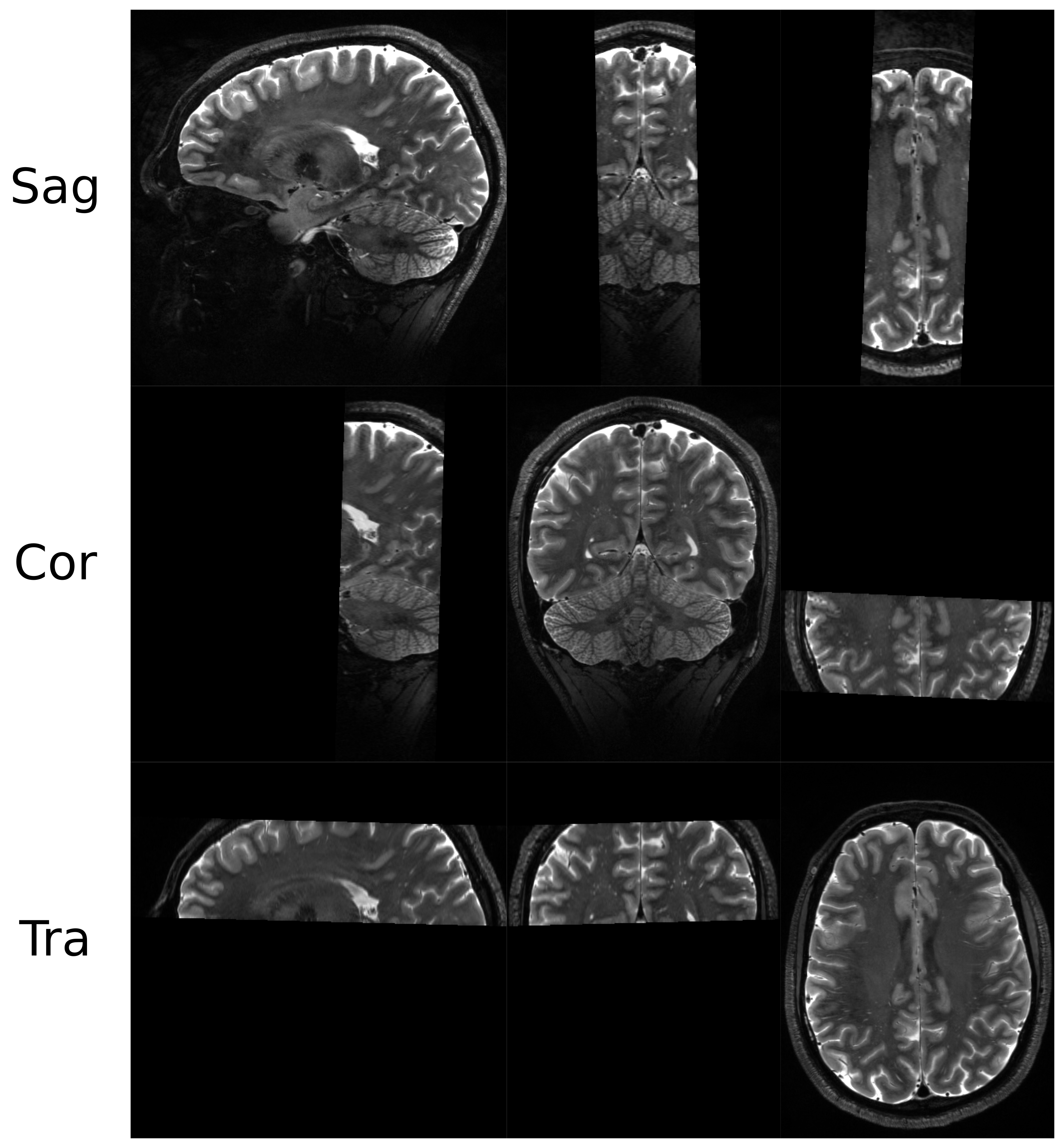


Figure S3: Boxplot illustrating inter-subject variability of different slice- (left) and slab-selective (right) pulses in Bloch simulations. Upper plots show the distribution of the mean FA across 10 subjects and lower plots show the FA-NRMSE. GRAPE pulses were designed as universal pulses (GRAPE and GRAPE ROI) or subject-tailored (ST) pulses (GRAPE ST and GRAPE ROI ST).


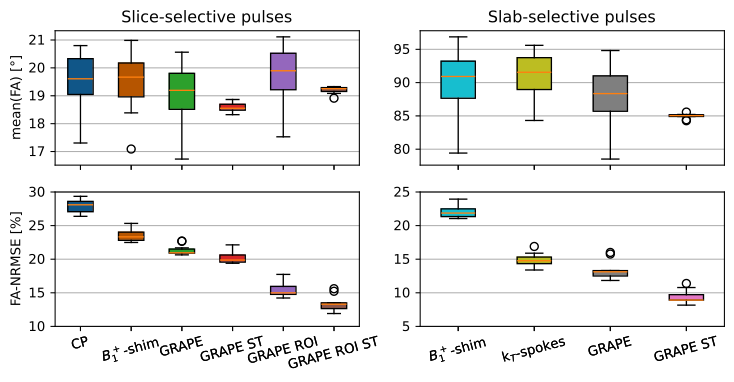


Figure S4: Bloch simulation results for slice-selective RF pulses. GRAPE pulses were optimized either on the whole brain (first two columns) or for the specific slice location (last two col-umns). Columns 1 and 3 show UPs. Columns 2 and 4 show subject-tailored (ST) pulses. For each case, the flip angle (FA) in the center of the respective slice and the integral over the slice in slice-selection direction (“slice integral”) were determined.

Figure S5: Flip angle maps from Bloch simulations and corresponding signal maps from extend-ed phase graph (EPG) simulations are shown for kT-spokes and GRAPE pulses (UP in the second column and subject-tailored (ST) in the third column).
